# Ammonium-to-sodium ion-exchange process at the interlayer of octacalcium phosphate†

**DOI:** 10.1039/d1ra07939e

**Published:** 2021-12-13

**Authors:** Yuki Sugiura, Yoji Makita, Masanori Horie

**Affiliations:** Health and Medical Research Institute, National Institute of Advanced Industrial Science and Technology (AIST) 2217-14, Hayashi-cho Takamatsu Kagawa Japan 761-0395 yuki-sugiura@aist.go.jp

## Abstract

Octacalcium phosphate (OCP) has been considered as the layer component of calcium phosphate, but whether it achieves the ionic-exchange ability of conventional layer components is unclear. As OCP is highly biocompatible, understanding its ionic-exchange properties would potentially expand its pharmaceutical and medical applications. Herein, we demonstrate that the substituted cations in ammonium (NH_4_)-substituted octacalcium phosphate (OCP-NH_4_) and sodium (Na)-containing ammonium phosphate solutions undergo ion exchanges with OCP interlayers. Replacing NH_4_^+^ with Na^+^ did not alter the crystal structure of OCP, confirming that a substituted cation exchange process similar to that in other layered compounds occurs in OCP.

## Abstract 2

Octacalcium phosphate (OCP) has been considered as the layer component of calcium phosphate, but whether it achieves the ionic-exchange ability of conventional layer components is unclear. In this study, we demonstrated the evidence of ionic exchange process at the interlayer of OCP.

## Graphical abstract

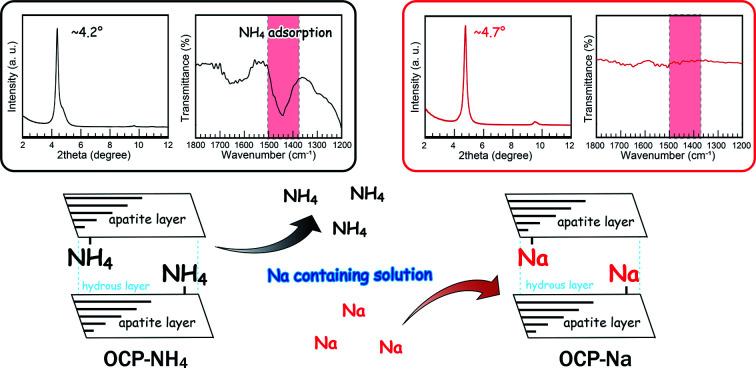

## Introduction

Octacalcium phosphate [OCP: Ca_8_H_2_(PO_4_)_6_·5H_2_O] is an attractive material in biomedical applications because its components—calcium (Ca), phosphate (PO_4_), and water (H_2_O)—are partially found in biological tissues and especially in bone.^1–3^ OCP has a layered crystal structure with relatively high stability and low environmental loading. Moreover, multiple ions and molecules, such as cations, dicarboxylates, and tris(hydroxymethyl)amino methane, can be substituted into precipitating OCP crystals without changing their crystallinity or crystal structure.^1,4–10^ These guest ions and molecules are mainly substituted in the hydrous layer of OCP, where they displace the Ca, PO_4_, and H_2_O constituents.^4,11–13^ In our previous studies on cation substitution under weakly basic solutions, we found that monovalent cations such as alkali metal ions and silver (mixture of states of Ag^0^ and Ag^+^) were substituted at the conjugated sites of P5 PO_4_, the root of the hydrous layer of OCP. When OCP was synthesized in the presence of NH_4_ and Na, the Na ions (rather than NH_4_) were preferentially substituted because their ionic radius is similar to that of Ca (Na^+^: 0.102 nm, NH_4_^+^: 0.175 nm, Ca^2+^: 0.100 nm).^14–16^

**Scheme 1:**
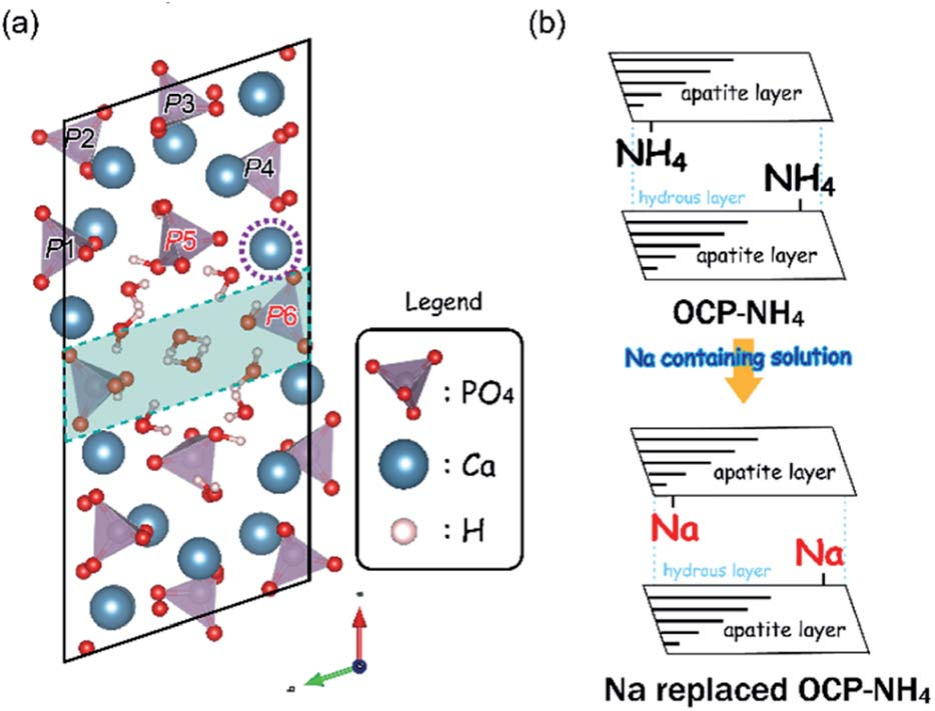
(a) Schematic of the OCP unit lattice-oriented toward the *c*-axis. The phosphate (PO_4_) groups are labeled P1–P4 and the green band highlights the hydrous layer. Purple broken-circled Ca indicates the replacement sites. (b) Schematics of the ion-exchange process of NH_4_ to Na in the OCP unit lattice.

Many examples of coexisting ions and molecules being substituted into the OCP unit lattice during the precipitation process have been reported. However, the process by which ions or molecules are substituted into the OCP unit lattice remains unknown. Considering the OCP crystal structure, the ion-exchange process of guest ions substituted in the interlayers of the OCP unit lattice probably mimics that of other layered materials such as clay minerals and micas (Scheme 1).^17–20^ In a case study of ammonium-substituted OCP (OCP-NH_4_) and Na-containing solutions, the present study examines the ionic-exchange process of guest ions substituted in the interlayers of OCP.

## Materials and methods

### Preparation of OCP-NH_4_

All reagents were purchased from FUJIFILM Wako Pure Chemical Industry Inc., Japan. The preparation method of OCP-NH_4_ was described in ref. 21. Briefly, 12.5 g monocalcium hydrogen phosphate monohydrate [MCPM: Ca(H_2_PO_4_)_2_·H_2_O] was immersed in 1 L of 1 mol L^−1^ diammonium hydrogen phosphate ((NH_4_)_2_HPO_4_) solution for three days at 40 °C. The treated samples were washed with distilled water several times, then dried in a dry oven at 40 °C overnight.

The reference material was OCP-Na, a conventional OCP formed under weak basic phosphate solutions as described in ref. 15. Briefly, 2.39 g calcium hydrogen phosphate dihydrate [DCPD: CaHPO_4_·2H_2_O] was immersed in 20 mL of 1 mol L^−1^ disodium hydrogen phosphate (Na_2_HPO_4_) solution for 1 day at 60 °C. The treated samples were washed with distilled water several times, then dried in a dry oven at 40 °C overnight.

### Ion-exchange experiment of OCP-NH_4_

The fabricated OCP-NH_4_ (0.4 g) was immersed in 40 mL of 1 mol L^−1^ (NH_4_)_2_HPO_4_ and 0–5 mol L^−1^ sodium nitrate (NaNO_3_) solution placed in 50 mL polypropylene centrifugation tubes at 40 °C. After closely packing, the vessels were shaked for 3 days at 150 rpm and 40 °C in a thermostatic shaker (BioShaker BR-23FP, Taitec Co., Japan).

The pH values of the suspensions were measured using a pH electrode (LAQUA ToupH 9615S-10D) connected to a pH meter (Horiba Co. D-72, Kyoto, Japan). The samples were then washed several times with distilled water to remove any residual immersion solution before being dried in a 40 °C oven overnight.

### Characterization

The crystallographic information of the samples was obtained in an X-ray diffraction (XRD) analysis (MiniFlex600, Rigaku Co., Japan) at an acceleration voltage and amplitude of 40 and 15 kV, respectively. The diffraction angle was continuously scanned over 2*θ* values ranging from 3° to 70° at a scanning rate of 5° min^−1^ for characterization and from 2° to 12° at 1° min^−1^ for crystallographic parameter analysis.

The chemical bonding scheme of the samples was characterized through Fourier transform infrared spectroscopy (FT-IR: Nicolet NEXUS670, Thermofisher Scientific Co., USA) using a triglycine sulfate detector (32 scans, resolution 2 cm^−1^) with an attenuated total-reflection GeSe prism. All measurements were conducted in the atmosphere.

After dissolving the samples in 2% HNO_3_ solution, the Ca, P(PO_4_), and Na concentrations in the samples were determined using inductively coupled plasma atomic emission spectroscopy (ICP-OES: 5110VDV, Agilent Technology Co., Japan).

## Results and discussion

The main aim of this study was to investigate the ionic-exchange phenomena of NH_4_ and Na in OCP crystals. Both the initial and final phases were required to be OCP. When OCP is immersed in aqueous solutions, it may convert to other phases such as hydroxyapatite [HAp: Ca_10_(PO_4_)_6_(OH)_2_] and/or DCPD *via* different phase-conversion processes such as dissolution and precipitation reactions. The dissolution and precipitation kinetics are mainly controlled by the ionic products and solubilities of the samples. The solubility difference between OCP and HAp was minimized under weak basic conditions. In addition, coexisting Ca and/or PO_4_ in the solution reduced the dissolving reaction of OCP, consistent with the ionic product hypothesis. We thus chose a weakly basic PO_4_ solution (1 mol L^−1^ (NH_4_)_2_HPO_4_) as the reaction solution.

**Fig. 1:**
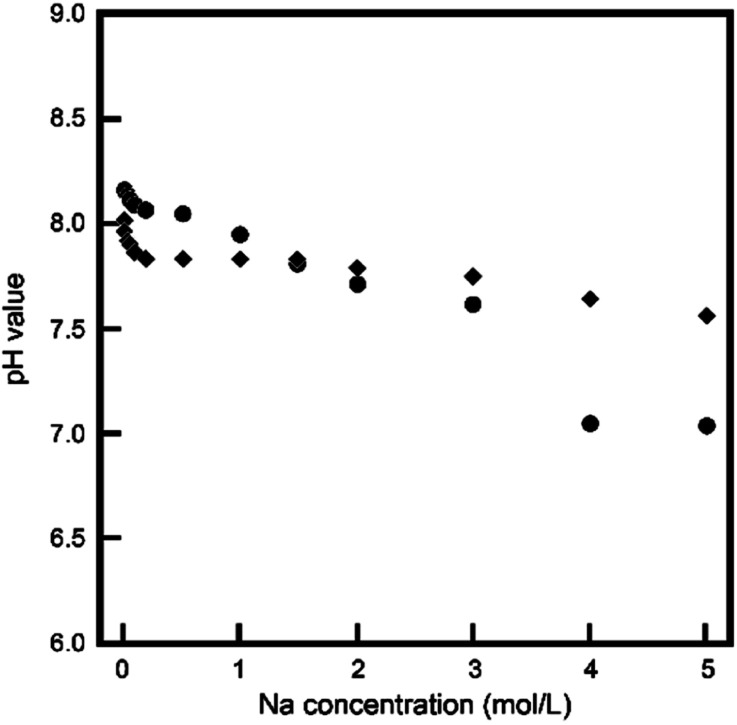
pH values of before (●) and after (◆) of treated solutions.

The pH value of the reacting solution mainly determined the process of the reaction. Fig. 1 plots the initial and final pH values of the reacting solutions as functions of Na concentration. Although both the initial and final pH values decreased slightly with increasing Na concentration, they remained in the weakly basic region.

**Fig. 2:**
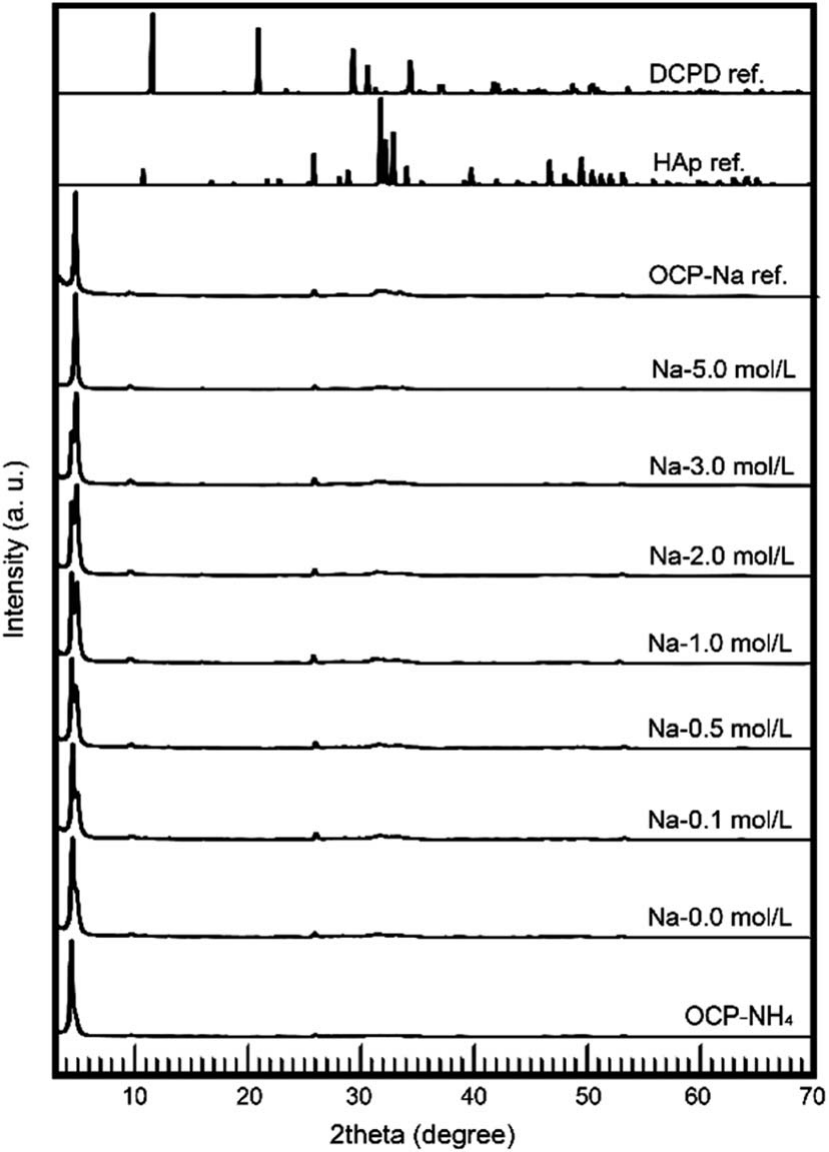
Wide-range XRD patterns of treated samples.

The most important factors of the OCP ionic-exchange process are the phases during the reactions. In this study, the phases were determined by XRD. Fig. 2 shows the wide-range XRD patterns of the samples. At all Na concentrations, the treated samples were monophasic OCP with no HAp or DCPD phases. The crystallographic phenomena in the OCP unit lattice were then subjected to further evaluation.

**Fig. 3:**
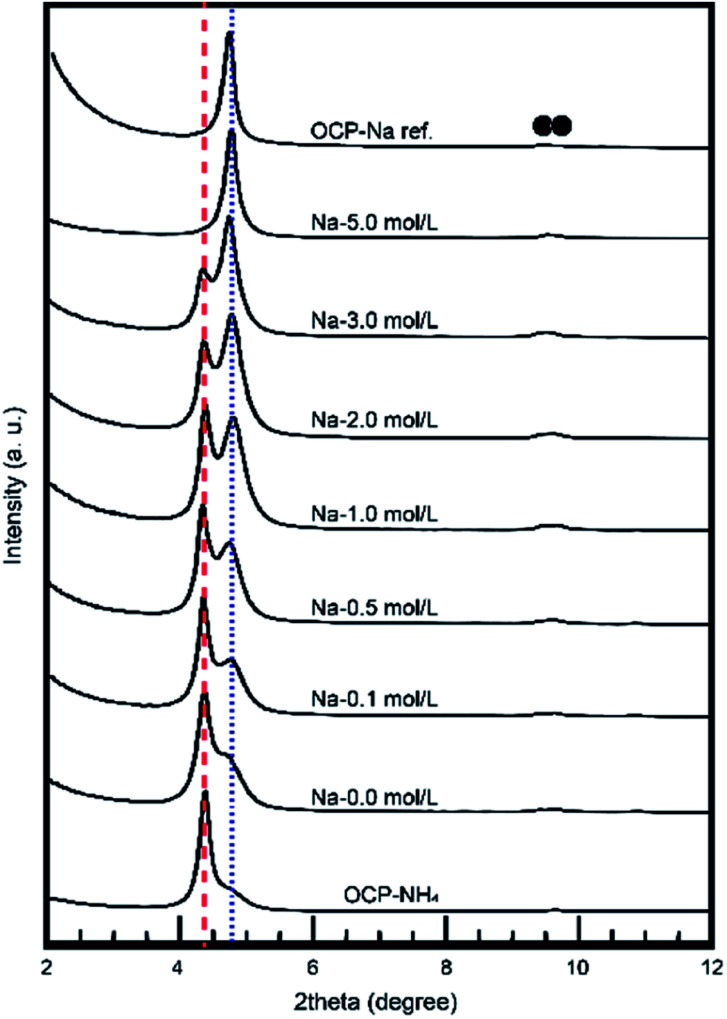
Small-angle XRD patterns of treated samples. ●: OCP, blue dot line: conventional OCP *d*(100) and, red broken line: OCP-NH_4_*d*(100)′.

**Fig. 4:**
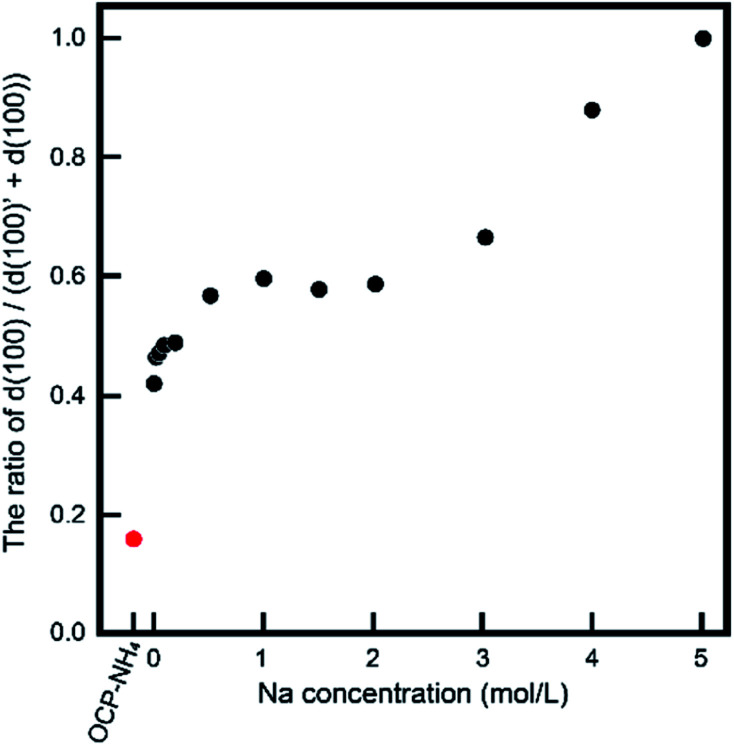
Relative ratio of OCP *d*(100)/[*d*(100) + *d*(100)′] *versus* Na concentration in solution.

The behavior of NH_4_-substituted OCP was easily detected as an extra peak at ∼4.2° [*d*(100)′] in the XRD spectrum. When Na was replaced at the same site (the P5 PO_4_ conjugating site of the OCP unit lattice), no significant peak shifts or extra peaks were observed and the usual OCP *d*(100) peak appeared at ∼4.7°. By estimating these peaks in the XRD pattern, we can elucidate the NH_4_ substitution mechanism and the Na-exchange degree in the OCP unit lattice. Fig. 3 magnifies the XRD patterns of the samples to highlight the main peaks and Fig. 4 plots the relative peak intensity ratio of *d*(100)′/(*d*(100)′ + *d*(100)) *versus* Na concentration. After immersion, the intensities of the *d*(100) peak of OCP-NH_4_ increased in all treated samples, indicating the development of a typical OCP unit lattice in the samples. Note that the *d*(100)′/(*d*(100)′ + *d*(100)) ratio of OCP-NH_4_ was significantly decreased even after immersion in the solution containing 0 mol L^−1^ Na. The *d*(100)′/[*d*(100)′ + *d*(100)] ratios in the treated samples increased with Na content up to 0.2 mol L^−1^ and then plateaued at ∼0.50 until the Na concentration reached 2.0 mol L^−1^. At this concentration, the *d*(100)′/(*d*(100)′ + *d*(100)) ratios of the treated samples began increasing again and the *d*(100)′ peak could not be detected at 5 mol L^−1^ Na.

The decreased *d*(100)′/(*d*(100)′ + *d*(100)) ratio of OCP-NH_4_ in Na-free solution was attributed to instability of OCP-NH_4_. When OCP-NH_4_ was immersed in different concentrations of (NH_4_)_2_HPO_4_ solution (0–1 mol L^−1^) as same manner of 1 mol L^−1^ (NH_4_)_2_HPO_4_ with NaNO_3_ solutions, its *d*(100)′ structure gradually decomposed over time. The decomposability of the *d*(100)′ structure of OCP-NH_4_ was an inversely proportional to (NH_4_)_2_HPO_4_ concentration (Fig. S1†).

**Fig. 5:**
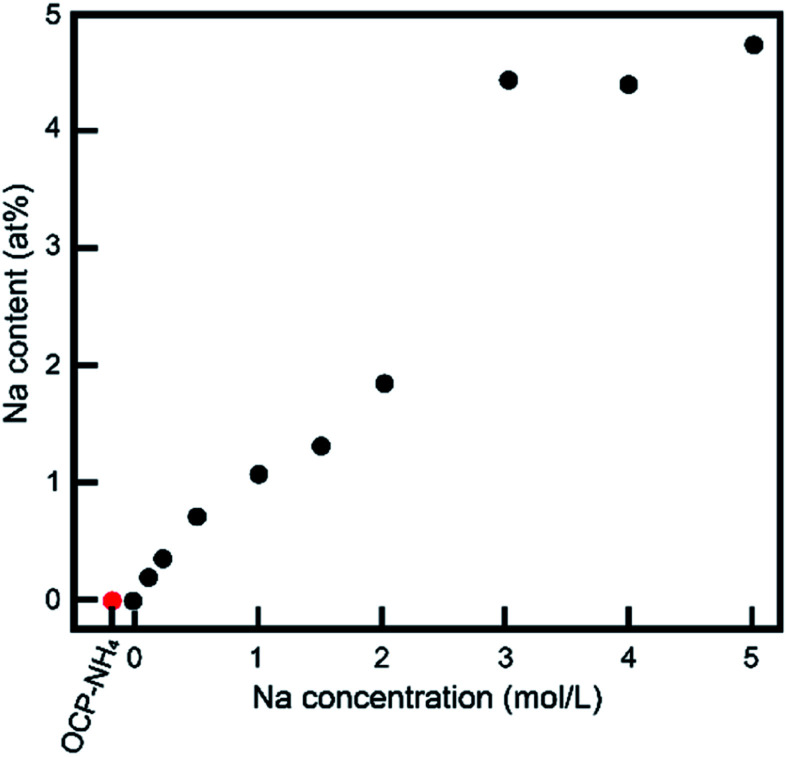
Na contents of samples as a function of Na concentration in solutions.

**Fig. 6:**
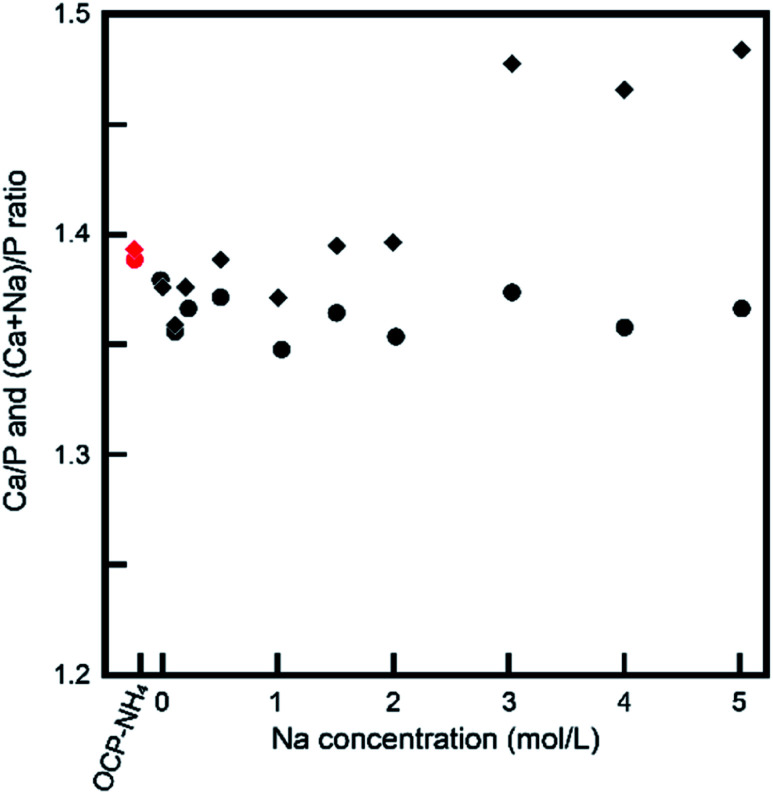
Ca/P ratio (●) and (Ca + Na)/P ratio (◆) of treated samples.

The XRD measurements imply an ionic exchange of NH_4_ with Na in the OCP unit lattice. The XRD data were validated in other chemical compositional analyses. Fig. 5 shows the Na contents in the samples determined by ICP-OES. The Na content in the treated sample increased as the Na concentration in the treatment solution increased up to 3 mol L^−1^, and plateaued thereafter. We also measured the Ca/P and (Ca + Na)/P ratios of the samples. Because the chemical composition might alter through dissolution and/or development of OCP processes other than NH_4_-to-Na ionic exchange, these ratios provide vital information about the crystallographic alternations during treatments. Before treatment, the Ca/P ratio of OCP-NH_4_ was 1.38. Fig. 6 plots the Ca/P and (Ca + Na)/P ratios of the samples *versus* Na concentration in the treatment solution. The Ca/P ratios of the treated samples trended nearly identically to those of OCP-NH_4_, but the (Ca + Na)/P ratios steadily increased with increasing Na concentration in the treated solution.

**Fig. 7:**
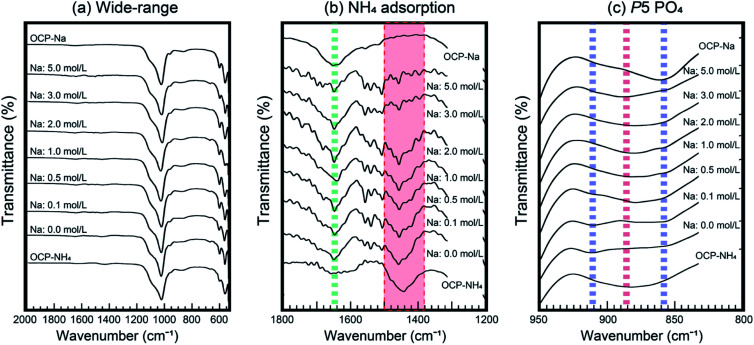
FT-IR spectra of the treated samples: (a) wide-range spectra; (b) region of NH_4_ adsorption bands, showing the HPO_4_ band (green broken line) and the NH_4_ adsorption band (red shaded region); (c) region of P5 PO_4_, showing the bands corresponding to cation adsorption of OCP-NH_4_ (red broken line) and OCP-Na (blue broken lines).

The ionic-exchange process of OCP-NH_4_ to OCP-Na was also evaluated by the spectroscopic method. Within the lowly symmetric crystal structure of OCP (*P*1̄), the vibrations of each component can be detected.^21,22^ The six major states of PO_4_ (P1–P6 PO_4_) in OCP all contain highly conjugated Ca ions.^15,23–25^ Our previous evaluations indicated that monovalent cations such as NH_4_ and Na can be substituted at the Ca site conjugated to P5 PO_4_, the root of the hydrous layer.^16,21^Fig. 7 shows the FT-IR spectra of the samples. Before immersion, the NH_4_ absorption band ranging from 1400 to 1500 cm^−1^ appeared in the spectrum of OCP-NH_4_. In the treatment solutions, increasing the Na concentration decreased the intensity of the NH_4_ absorption band. Above 3 mol L^−1^ NaOH, the NH_4_ absorption band disappeared. This observation was consistent with the XRD analysis. However, at the P5 PO_4_ site, numerous bands related to Na-substituted OCP emerged with increasing Na concentration in the treatment solution, but the band corresponding to OCP-NH_4_ remained visible at high Na concentrations.

**Scheme 2:**
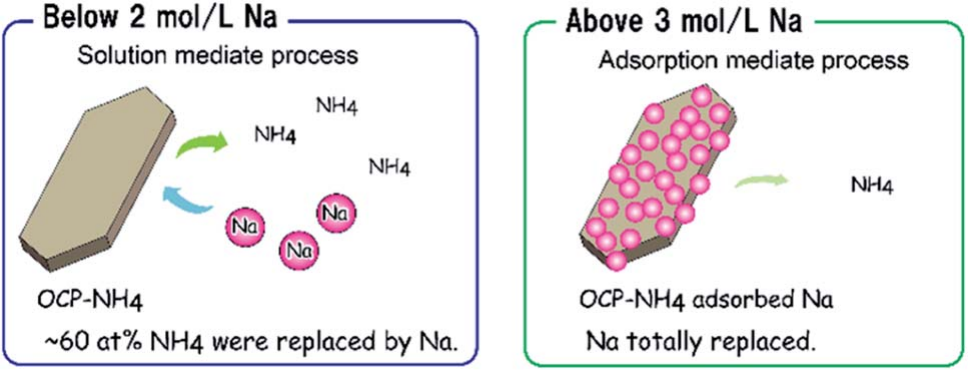
Schematic of the ion-exchange process of OCP-NH_4_ in systems with different Na concentrations.

The obtained results provided clear evidence of the ion-exchange process at the NH_4_-substituted sites of OCP-NH_4_ in Na solution. The NH_4_-exchange degree of OCP-NH_4_ was controlled by varying the Na concentration in the treatment solution. However, the ionic-exchange process in the OCP unit lattice required a higher Na concentration than direct precipitation.^16^ The above chemical composition analysis indicated that the threshold value of Na intercalation into OCP-NH_4_ depended on the Na concentration in solution. Below 2 mol L^−1^ Na, the ion-exchange process of OCP-NH_4_ dominated and the *d*(100)′/(*d*(100)′ + *d*(100)) of OCP-NH_4_ reached ∼0.6. Above 3 mol L^−1^ Na in solution, the ionic-exchange process apparently reached the threshold in simple exchange and the Na contents in the samples no longer increased. Thus, it was suggested that Na adsorption simultaneously occurred on the sample surfaces with ion exchange process. It was also suggested that Na adsorption onto OCP-NH_4_ influences further ion-exchange processes. The expected exchange phenomena are summarized in Scheme 2.

Heretofore, although various molecules and cations can be substituted in the OCP unit lattice, ordinary ionic-exchange properties of OCP have not been reported because OCP exists in a metastable phase.^2,26^ However, we verified that OCP, like other layered components (clay minerals and layered double hydrates), possesses ionic-exchange properties under optimal settings.^17–20^ This phenomenon can be exploited in new clinical usages of OCP, especially, in combinations of medical products. For example, a controlled burst of medical drugs could be released from substituted interlayers of the OCP unit lattice.

## Conclusions

We demonstrated the ionic-exchange properties of OCP in OCP-NH_4_- and Na-containing solutions, which are weakly basic phosphate buffer solutions. NH_4_-substituted OCP was gradually replaced by Na as the ionic exchange proceeded. The degree of Na exchange was comparable with the Na concentration in the reaction solution.

## Conflicts of interest

There are no conflicts to declare.

## Supplementary Material

RA-011-D1RA07939E-s001
