# Supplementary material for: Ammonium-to-sodium ion-exchange process at the interlayer of octacalcium phosphate
Source: RSC Adv. 2021 Dec 13;11(62):39503–7. doi: 10.1039/d1ra07939e (PMC9044421; doi:10.1039/d1ra07939e)
Supplement: RA-011-D1RA07939E-s001 [file RA-011-D1RA07939E-s001.pdf]

Electronic Supplementary Material (ESI) for RSC Advances.
This journal is © The Royal Society of Chemistry 2021

Supporting Information: Ammonium-to-sodium ion-exchange process at the interlayer of octacalcium phosphate

Yuki Sugiura, Yoji Makita, and Masanori Horie

Figure S1.

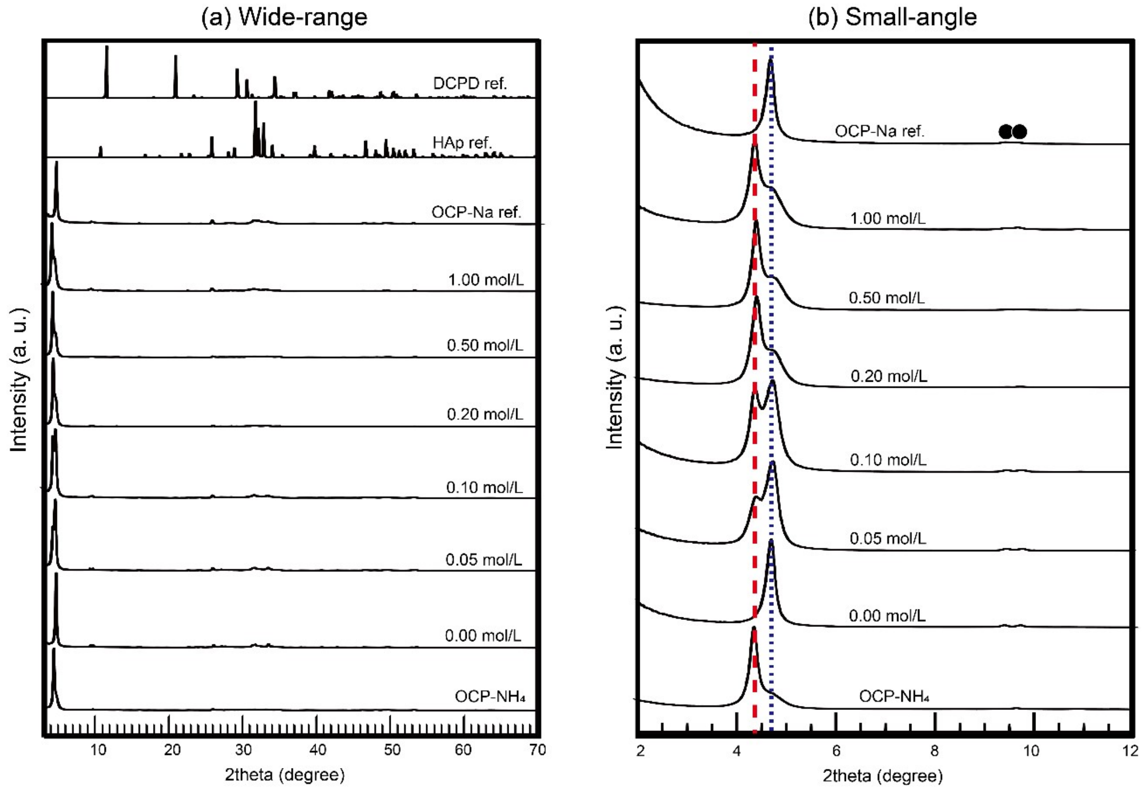

Figure S1. Wide-range (a) and small-angle (b) XRD patterns of treated samples in different $(NH_4)_2HPO_4$ solutions. ●: OCP, blue dot line: conventional OCP d(100) and, red broken line: OCP-NH_4 d(100)'.
